# Cooling System with PCM Storage for an Office Building: Experimental Investigation Aided by a Model of the Office Thermal Dynamics

**DOI:** 10.3390/ma14061356

**Published:** 2021-03-11

**Authors:** Jarosław Karwacki

**Affiliations:** Institute of Fluid Flow Machinery, Polish Academy of Sciences, Fiszera 14 Str., 80-231 Gdańsk, Poland; jaroslaw.karwacki@imp.gda.pl

**Keywords:** phase change material (PCM), thermal energy storage (TES), energy management, mathematical modelling, load shifting

## Abstract

The application of energy storage filled with phase-change material (PCM) is recently increasingly considered in active cooling systems. Such a design offers a higher density of thermal energy accumulation when compared with water storage. However, the optimum use of PCM storage is possible when its dynamic characteristics during the loading and unloading process are well recognized. Due to the complexity of the interaction between all elements of the heating/cooling system, a theoretical estimation of the profits is hard to perform in a reliable way. This is a significant problem at the design stage of the installation. In order to solve this problem, a laboratory experiment supported by a simulation was performed. The main aim of the experiment was to understand how the storage filled with the PCM in real-like conditions works. A test stand was made to investigate the effect of this solution on a reduced scale of 1:10. The PCM tested was RT15, a commercially available material that melts in the temperature range of 10–17 °C. The main parts of the stand are a chiller, an electric heater and thermal energy storage. The first two elements allowed a simulation of the thermal properties of the heat receiver, and their operation depended on the results from the numerical calculations. A lumped parameter model was used in mathematical description of the office building and its cooling system. The heat capacity of the system components as well as heat losses to the ambient environment were taken into account. The obtained results allowed the optimization of the control procedure and proved the validity of the applied investigation methods. This study confirmed the possibility of testing thermal energy storage with phase change material in real-like conditions.

## 1. Introduction

Sensible heat storage using water is the most widely used technology of energy storage; however, nowadays phase change materials (PCMs) are more frequently utilised in the low and high temperature applications [1,2]. The PCM heat storage utilises the process of the phase transition between a solid and a liquid to store thermal energy. In the simplest approach, the heat transfer process during melting and solidification proceeds at a nearly constant temperature. In a narrow range around the phase change temperature, the PCM stores or releases large quantities of thermal energy. In practice, due to the temperature gradient in the material, many PCMs change the phase over a certain temperature range. Moreover, an enthalpy–temperature characteristic h(T), usually used for the performance calculations, shows hysteresis [3,4]. That is why a theoretical estimation of the profits resulting from the use of PCMs in a real application is difficult to perform in a reliable way. In order to propose a strategy for dealing with the estimation problem, the present study has confirmed the possibility of testing the LHTES (Latent Heat Thermal Energy Storage) in conditions close to the real application. In the author’s opinion, such an approach can reveal information not only about the real storage capacity of LHTES but also about its dynamic characteristics during the loading and unloading process.

Due to the rapid increase in energy demand in the construction industry, PCMs are the most widely used in this area. They are often considered as additives to mortar or concrete [5,6]. Such a solution can reduce the indoor temperature fluctuation, leading to improved human comfort and decreasing building energy consumption. The use of PCMs in structural elements can be considered a passive method of improving the energy efficiency of a building. Another solution is to use the element with the phase change material as part of the cooling or heating system [7,8] This solution, called an active cooling system, is analyzed in the presented work.

**Figure 1:**
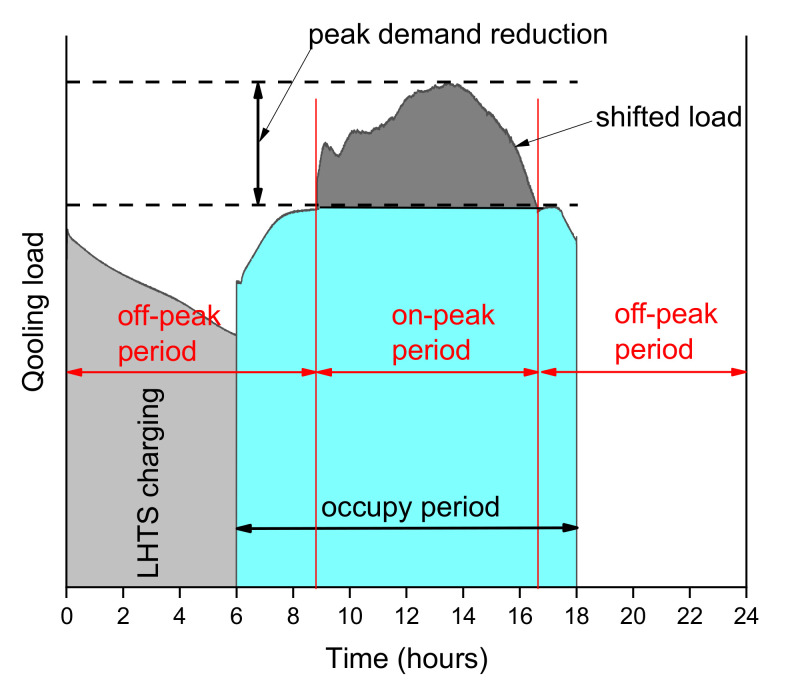
Building cooling load profiles for one day, illustrating the load shifting strategies.

The heat storage with the phase change material (LHTES) has been used in the refrigeration and air conditioning applications for many years. The most widely known domain of LHTES application is the peak demand management in commercial buildings [9,10]. Most frequently, ice banks (ITES) with a chiller are used in the cooling and air conditioning systems to reduce the thermal energy load during on-peak hours. They work during the night to charge the ITES, when the energy costs are low, and use accumulated cooling energy during the day. In Figure 1, a schematic diagram of load shifting control is presented. In a typical situation, without heat accumulation, the chiller plants and the refrigeration systems tend to be oversized concerning their maximum design load conditions. This certainly leads to higher energy consumption than in a properly sized chiller plant. The ice bank systems allow to use the cooling aggregate with a much smaller power than the peak load of cooling energy appearing during its operation. In many cases, it allows a reduction in the overall operating and investment costs [11,12].

In the cooling applications, water is the most popular PCM, but the use of other PCMs is also increasingly considered [13]. This is due to the possibility of choosing virtually any phase transition temperature in the range of typical temperatures used in air-conditioning systems. The possibility to raise the heat transfer fluid (HTF) temperature and thus the evaporation temperature allows an increase in the energy efficiency, and thus a reduction in the costs of operation. In the cooling peak demand management system, the basic task of the TES is to store the energy from the chiller to be later used as an additional cooling source. A full cycle involves the processes of charge, storage and discharge. Usually, it is realised by the intermediate heat transfer fluid. The energy obtained from the TES system during discharge not only depends on how the energy was supplied into the system during the charging process, but also depends on the behaviour of the TES system during the storage process.

In active peak load shifting systems, the optimal operation of the heat storage with PCM involves not only the selection of a suitable material, but also proper control of its thermal loading and unloading. Optimum operation of the PCM storage requires recognition of its dynamic characteristics. The literature presents a number of LHTES experimental analyses in various dynamic conditions. Most of them were performed in a small, laboratory scale. This type of experiment allows an understanding of the heat transfer processes during the melting and the solidification for the particular shapes of the heat transfer area [14]. Nevertheless, experimental studies on a larger scale are needed for guidance on the practical use of PCMs in active peak load shifting systems. In general, an experimental study carried out in the laboratory conditions allows one to obtain more accurate and reliable measurement results [15,16,17]. Relatively rarely, the LHTES is installed as a part of the production line and tested under real operating conditions [18]. In such a situation, it usually does not influence the test condition. Despite this, LHTES charging and discharging characteristics could be defined.

Concerning the LHTES, there are a few methods to determine its heat storage capability and the dynamic response. The most common measurement technique is based on an instant change of inlet HTF temperature (step function) [15,16,19]. In such experiments, the HTF input temperature is instantaneously set at the predetermined value, and then the evolution of output temperature is recorded. The range of inlet temperature change is appropriately selected to include the phase transition interval of the PCM used. For example, Nuyten et al. [15] carried out the experiments with the steps ±5 °C, ±10 °C and ±15 °C above and below the melting point. In some cases, a sudden change in the HTF temperature can simulate the operation of a real system. J. Porteiro at al. [20] incorporated three different types of encapsulated PCM in the hot water storage tank. The used test methodology was intended to simulate the real response of a domestic hot water system consisting of one large demand and two short demand.

The pure step function test is usually difficult to carry out because thermal inertia of the installation. One of the ways is to use two separate storage vessels containing HTF at different temperatures [15]. Such a solution allows to obtain the required dynamics of temperature changes; however, due to the limited volume of the tanks, the experiment time is finite. This problem does not exist in systems with a closed HTF circuit. Generally, the step change of the set HTF inlet temperature does not result in a sudden reaction of a closed HTF cycle. Instead, sometimes it induces a fluctuation of the inlet temperature or the temperature profile change is not sharp enough [14,16]. In some cases, it significantly influences the overall quality of experimental results.

In most of the published experimental research, the attention is focused on determining energy stored in the TES under full capacity loads. Sometimes, an experiment was also performed to analyse the storage period in a system with LHTES working under partial load operating conditions [15,21]. Such tests aim to simulate real working conditions and obtain useful information for future application. In the recent study by Gasia et al. [21], great attention was paid to the impact of the storage period on the subsequent discharging process. It was worth noticing that depending on the level and the dynamics of the charging process, the PCM was not completely melted in some regions of the TES container. In the experiments, different levels of temperature homogenization were observed. During the storage period, the PCM temperature showed a tendency to homogenise. It was observed, that for that particular TES (99.5 kg of high density polyethylene), the storage period over 30 min slightly affected the temperature and heat transfer profile. Charging and discharging rates were also calculated.

Due to the complexity of the cooling system elements, a theoretical estimation of the profits resulting from the use of the LHTES is difficult to perform in a reliable way. Analysis based on the experiments with the typical thermal forcing could provide some information about heat transfer dynamics in thermal energy storage. However, in many cases, it seems not to be enough. From the literature review, it can be observed that there is still a lack of experimental results that evaluate the effect of the storage period duration in cyclic processes. Furthermore, none of these papers presented a systematic methodology for the determination of the dynamic characteristics.

There is a lot of experimental work considering the application of PCM, but most of them concern the use of PCM in passive systems, mostly to increase the heat capacity of structural elements. Only a few experimental works focus on the use of PCM in active systems, and most of them use simple thermal forcing (line). Hardly any of the works deal with the dynamics of temperature variations occurring in real cycles. Continuing the previous work [17], this paper presents a concept of experimental testing of the operation of LHTES in real-like conditions. This approach allows for a more reliable assessment of the thermal capacities applying to specific store, not just an estimate based on the manufacturers’ material data. Therefore, an attempt to test the heat storage with thermal excitations close to the real system presented in this study can be regarded as a novelty. It should also be emphasized that the presented method is based on a modelling-assisted experiment.

The aim of the presented research is to evaluate the thermal energy capacity and the charging/discharging rate of a thermal energy storage tank working in real-like conditions and to verify a prototype construction of LHTES.

## 2. Materials and Methods

### 2.1. General Assumptions

**Figure 2:**
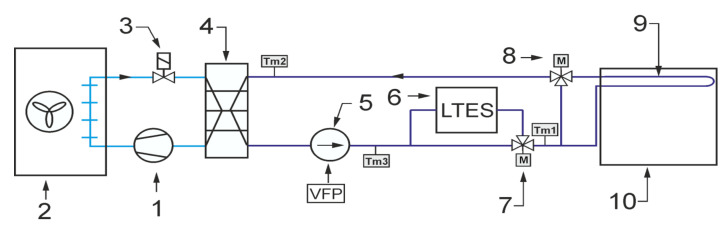
General scheme of the emulated office building cooling system configuration: **1**—compressor, **2**—condenser, **3**—expansion valve, **4**—evaporator, **5**—circulating pump, **6**— Latent Heat Thermal Energy Storage (LHTES), **7**—3-way mixing valve, **8**—3-way valve, **9**—ceiling heat exchanger, **10**—office building (cooling load).

As already mentioned, the main purpose of the experimental work was to check the operation of the storage with PCM in conditions close to real use. In particular, the experiments were intended to confirm the possibility of carrying out such research on an existing test stand. It was assumed that the operation of the office building’s cooling system could be emulated experimentally by a simple chiller and an electric heater. During the experiment, both the office building and the cooling system were simulated using a mathematical model. The modeled receiver and chiller provided the temperature forcing for the tested LHTES that closely corresponded to the real-life operating conditions. The general scheme of the system configuration that was studied is shown in Figure 2. It was intended to cool down the office building using a typical compressor chiller combined with an energy storage tank. The task of the experimental stand was to provide storage inlet parameters corresponding to working conditions of the real system.

The typical cooling system is designed to maintain the room temperature *T_i_* in the comfort range during the occupied time span. Due to the complexity of the analysed problem, a simple configuration of the indirect cooling system was selected. The cooling system can be divided into two circuits. The first one is a refrigeration cycle that consists of a compressor 1, air cooled condenser 2, throttling valve 3 and evaporator 4. It was assumed that a typical reciprocating compressor would be used with well-known performance characteristics. Therefore, it is possible to determine the cooling capacity for the assumed condensing and evaporating temperature values. The condensing temperature *T_c_* was assumed to be 10 °C higher than the ambient temperature *T_a_*. The evaporating temperature *T_e_* was assumed to be 9 °C lower than HTF inlet temperature *T_m2_*. This corresponds to the operation of the refrigeration unit with superheating by 6 °C. It was assumed that the refrigeration unit works only in ON/OFF mode.

The second circuit, i.e., the heat transfer fluid cycle, consists of a circulating pump 5, thermal energy storage with PCM 6, mixing motor operated valve 7, 3-way motor operated valve 8 and indoor heat exchanger 9. It was assumed that the office cooling system uses the technology of cooled ceiling with capillary tube mats [16]. The capillary tubes have an outer diameter of only 3.35 mm and a wall thicknesses of 0.5 mm. Therefore, the capillary tube mats can be installed very close to the ceiling surface. As a result, the overall thermal resistance is low. The narrow spacing between the capillary tubes ensures uniform surface temperatures with high energy efficiency. This results in very short response times for the cooling surfaces and a very comfortable temperature distribution in the room. Considering energy efficiency, the main advantage of using a surface cooling system is the increase in the feed water temperature. Usually, in the typical office cooling system, the feed water is 7 °C, which causes the evaporation temperature to be around 0 °C. The surface cooling system allows to rise this temperature up to 18 °C, which increases the evaporating temperature and significantly reduces the chiller energy consumption.

**Figure 3:**
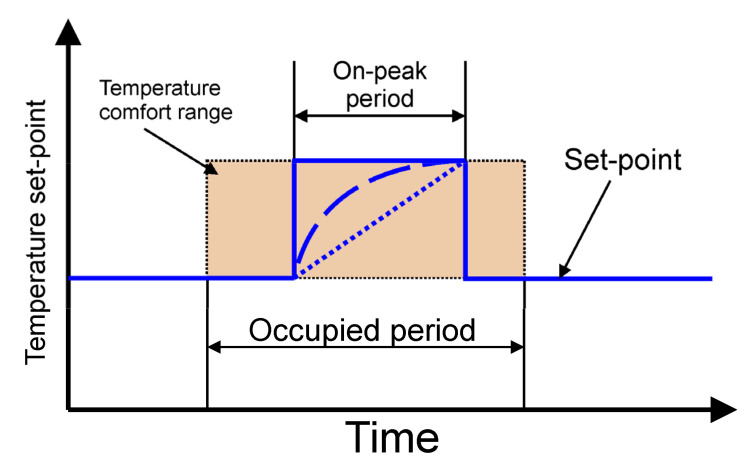
Hourly profiles of the set-point temperature.

The following describes how the simulated cooling system for the office building works. It was assumed that the chiller cooling capacity is not sufficient to secure the proper indoor temperature during whole day. For this reason, energy is stored in TES after a period of cooling demand. In this case, the valve 7 ensures that all liquid flows through the TES. The position of the valve 8 allows bypassing the office building heat exchanger. During the day, all HTF flows through the cooled ceiling. The task of the regulation system is to control the mixing valve 7 to maintain the indoor temperature in the comfort range. Most often, a proportional-integral (PI) controller is used for this purpose. The controller determines the mixing valve position. In PI control, it is necessary to specify a process variable *T_i_* and setpoint temperature *T_set_*. To keep office temperatures in a comfortable range, different control strategies based on the hourly setpoint *T_set_* temperature scheduling can be adopted [10,11]. In this case, only the step strategy was tested experimentally (Figure 3).

### 2.2. Methodology

One of the main goals of this work was to develop and validate a methodology for laboratory testing the thermal energy storage with PCM in close to real dynamics conditions. Many methods are used to determine the overall and partial thermal capacity of storage [21,22] The most common measurement technique is based on a step change of inlet HTF temperature. For LHTES, the range in which the temperature varies includes the phase change temperature. In such experiments, the input temperature of the HTF is rapidly set at the desired value, and thereafter the evolution of output temperature is recorded. In such a case, the results obtained may not reflect the actual heat capacity of the storage and the dynamics of the charging and discharging process. In the presented work, it was assumed that LHTES will be tested in conditions close to real use. This means that hourly profiles reflecting the thermal load of the building will be used for testing. It was assumed that the inlet LHTES parameters will be calculated from outlet LHTES condition resulting from the mathematical model. The model should sufficiently describe the actual heat load and cooling system. Experimental tests were carried out on a prototype LHTES. The storage was made in laboratory scale using a 40 dm^3^ vessel with a spiral-shaped coil. The vessel was filled with 27.9 kg of PCM, the commercially available material RUBITHERM RT15 (Rubitherm Technologies GmbH, Berlin, Germany) with the heat capacity of 150 kJ/kg in the temperature range of 7–22 °C [18]. The procedure for preparing and carrying out the experiment is described below.

At first, the heat capacity of the used LHTES was estimated. Assuming that the other elements of the tank have a negligible heat capacity, the amount of heat accumulated in the temperature range from 7 °C to 17 °C is 1.25 kWh. Calculations were made on the basis of characteristics PCM RT15 provided by the manufacturer [23]. The geometry of the heat transfer surface used in the LHTES can be treated as a segment of a larger tank. It was assumed that it is possible to perform a laboratory experiment on a scale of 1:10 with respect to thermal loading. This is associated with the assumption that the flow of liquid in the laboratory experimental system must be multiplied by a factor of 10 to obtain the results corresponding to the cooling system serving a (virtual) office building simulated by the calculation model. That means that the heat capacity of the full scale LHTES is 12.5 kWh. Please note that in the rest of the paper, all results relate to the 1:10 scale factor. It was assumed that the cold store will cover 30% of the total heat demand in the peak period. It follows that the total cooling demand in the same time should be around 41.7 kWh.

**Figure 4:**
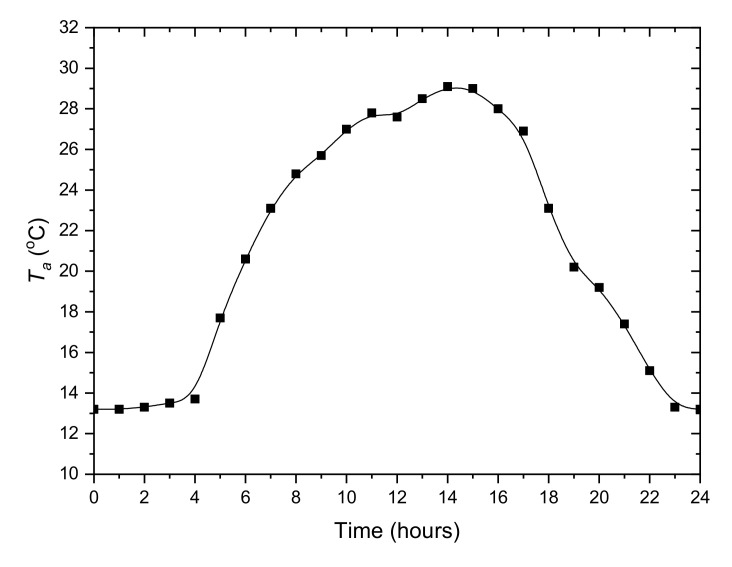
Hourly profile of the ambient temperature adopted for modelling.

**Figure 5:**
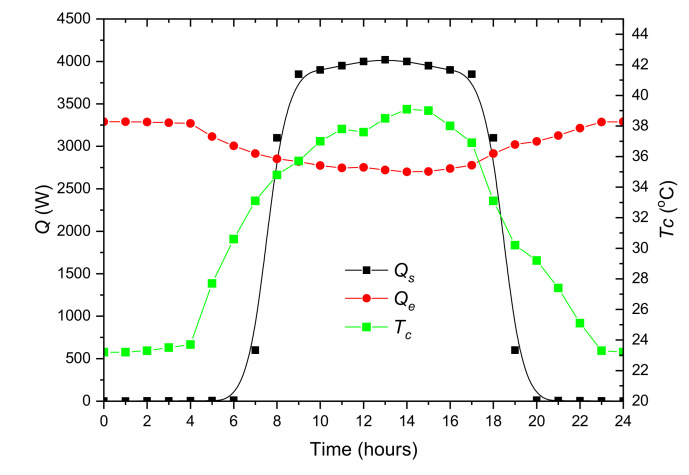
Hourly profiles of the office building internal cooling load *Q_s_*, compressor unit cooling capacity *Q_e_* and condensing temperature *T_c_*.

Subsequently, the daily cooling demand profile was determined. The air temperature measured on 1 July 2019 by the weather station Lufft WS700-UMB (G. Lufft Mess- und Regeltechnik GmbH, Fellbach, Germany) installed at the IMP PAN building in Gdańsk, Poland was selected. This was the day with the highest average temperature of the year. The hourly ambient temperature profile is presented in Figure 4. The resolution of the data describing internal heat sources and ambient temperature is 1 h. To determine the total cooling load of internal heat sources in the office, it was assumed that the average power of the surface cooling system is 40 W/m^2^ [24]. For a room dimension of 10 × 10 m assumed in the model, it totals 4000 W. The adopted hourly profile of heat load from internal sources is shown in Figure 5. In addition, the figure shows the cooling capacity of the compressor unit calculated for the constant evaporation temperature 9 °C and the outside temperature *T_a_* (Figure 4). Both the cooling capacity of the chiller and the overall cooling demand of the office depend on the ambient temperature. The preliminary analysis shows that during peak load periods the building energy consumption will be higher than the chilling power. It should be remembered that in the real system, the evaporation temperature is variable and the cooling capacity will be different from that presented in the Figure 5. More accurate calculations, taking into account other heat sources, will be performed based on the solution of lumped model equations. In the calculations, continuous profiles obtained from spline data interpolation were used. The profiles are marked on the Figure 4 and Figure 5 with a solid line.

In the next step, the refrigeration compressor unit with well-defined characteristics was selected. A good approximation of the unit performance can be derived from the characteristics taking into account the condensing and evaporating temperatures. The compressor’s performance declarations in Europe are regulated according to the standard EN 12900 [25]. The declared performance according to this standard shall comprise the refrigerating capacity and the absorbed power at dew points for evaporating and condensing temperatures. The characteristics are described by a polynomial equation in the following general form:

(1) $$y=c_{1}+c_{2}T_{e}+c_{3}T_{c}+c_{4}T_{e}^{2}+c_{5}T_{e}T_{c}+c_{6}T_{c}^{2}+c_{7}T_{e}^{3}+c_{8}T_{e}^{2}T_{c}\;+c_{9}T_{e}T_{c}^{2}+c_{10}T_{c}^{3}$$

where

*y*—compressor refrigerating capacity or absorbed power;
*T_e_*—evaporating temperature at suction dew point;
*T_c_*—condensing temperature at suction dew point.

Based on the BITZER software [25], the semi-hermetic reciprocating compressor was selected. It was assumed that during peak hours the compressor performance must cover 70% of the building’s cooling demand. This means that a compressor with a cooling capacity of around 2800 W is required. The following nominal selection parameters were assumed:

1. Compressor without capacity control;
2. Dry-cooler without capacity control;
3. Refrigerant R134a;
4. Suction gas superheating 6 °C;
5. Liquid subcooling 0 °C;
6. Ambient temperature 28 °C;
7. Condensing temperature 38 °C (10 °C above the ambient temperature);
8. Ceiling heat exchanger supply temperature 18 °C;
9. Evaporating temperature 9 °C (9 °C bellow supply temperature).

Based on the above assumptions, the BITZER 2KES-05Y (BITZER Kühlmaschinenbau GmbH, Sindelfingen, Germany) compressor model was selected. The compressor’s nominal cooling capacity is 2740 W (a value close to the requirement), and the energy efficiency ratio EER is 4.2. The capacity and the electric power polynomial (1) coefficients *c* are presented in Table 1.

**Figure 6:**
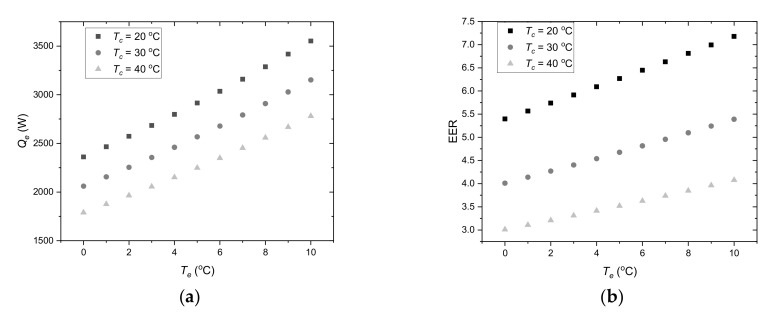
Compressor chiller load (**a**) and energy efficiency ratio (**b**) as a function of the evaporating temperature *T_e_*.

Considering the ambient temperature range (Figure 4), it can be predicted that the condensing temperature is in the range of 20 °C to 40 °C. In accordance with the assumptions, for the cooling system supply temperature 18 °C, the evaporation temperature is 9 °C. During LHTES charging, the heat transfer fluid temperature will be lower and, of course, evaporating temperature could drop below 9 °C. For further analysis, an evaporation temperature range from 0 °C to 10 °C was assumed. Compressor cooling capacity (Figure 6a) and energy efficiency ratio (Figure 6b) were determined for these parameters. Based on these characteristics, it is possible to specify guidelines for controlling the cooling system using LHTES. It can be seen that the cooling capacity *Q_e_* increases with increasing evaporation temperature and thus the HTF temperature. This means that in the off-peak period, the control system should maintain the HTF temperature as high as possible. In this case, the upper temperature is limited by the need to maintain the indoor temperature *T_i_* in thermal comfort range. The second limitation results from the maximum evaporating temperature for the compressor, which is 10 °C. From the energy efficiency point of view, the evaporation temperature should also be as high as possible during LHTES charging. The evaporation temperature depends primarily on the heat transfer conditions, which in turn depend on many parameters such as LHTES design, type of PCM and HTF parameters. This is a complex problem, which is difficult to analyse and optimize based only on theoretical considerations.

The second important parameter that significantly affects the energy efficiency of the cooling system is the condensing temperature. It can be seen (Figure 5) that the cooling capacity *Q_e_* increases with decreasing condensing temperature *T_c_*. When using a dry-cooler, this temperature depends primarily on the ambient temperature. Due to this fact, the chiller’s cooling capacity is not sufficient enough during the peak period (Figure 5), and the chiller works at maximum cooling capacity. During this period, it is not possible to use the *Q_e_* dependence on *T_c_* to optimize the cooling process. The LHTES charging process can be carried out at any time outside the peak period. By charging the storage during the low ambient temperature period, the total electricity consumption can be minimized. However, in this case, due to the complexity of the problem, the optimization process is also complicated. In the author’s opinion, the approach proposed in this work may be a good way to check and optimize the control system before its implementation in a real system.

The experiments comprised one type of simulation the cooling system control strategy (Figure 3). The step control strategies carried out experimentally for the present study were repeated three times to demonstrate the repeatability of the methodology and experimental results. The measurement results obtained in the third cycle were selected for further analysis.

### 2.3. Computational Model of the Cooling System and the Office Building

**Figure 7:**
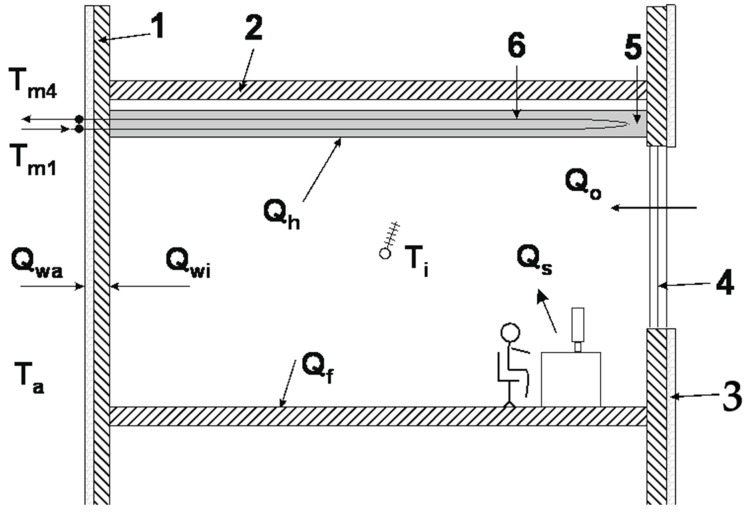
The schematic view of model office building: **1**—outer wall, **2**—ceiling/floor, **3**—insulation, **4**—window, **5**—cooling ceiling, **6**—capillary tube mats.

Thermal dynamics of the cooling system consisting of the compressor chiller, cold receiver (office building), thermal energy storage system and control valve were analysed by means of a 0 D mathematical model based on the heat balance equations. In the model, the heat capacities of the liquid loop and all the above mentioned system elements were taken into account. For simplification, it was assumed that heat exchange occurs by conduction and convection only. Thermal radiation was not considered. It was assumed that the analysed room space was located between two identical offices. This means that the temperatures on both sides of the partition (ceiling/floor) are the same. It was assumed that the building structure was made from modern technology and was based on prefabricated multi-layer walls [26]. Moreover, the calculation assumed that the influence of inner walls was negligible. Figure 7 shows the building elements included in the mathematical model. Detailed information about the items is provided in Table 2.

The proposed simplified numerical model of the office building describes heat transfer and accumulation between the indoor air and the wall, as well as the heat transfer to or from the surroundings. The equations of the numerical model are based on a balance of the heat flux accumulated in the material of a mass M and specific heat c, and the difference between the supplied heat flux ${\dot{Q}}_{in}$ and the dissipated heat flux ${\dot{Q}}_{out}$. This balance can be written in the following general form:

(2) $$\dot{Q}=Mc\frac{dT}{d\tau }=\sum {\dot{Q}}_{in}(\tau )-\sum {\dot{Q}}_{out}(\tau ),$$

where *τ* denotes time. Basing on Equation (2), balance equations for each material that accumulates heat in the system can be derived, namely,

Indoor air:

(3) $$M_{i}c_{i}\frac{dT_{i}}{d\tau }={\dot{Q}}_{h}+{\dot{Q}}_{wi}+{\dot{Q}}_{f}+{\dot{Q}}_{o}+{\dot{Q}}_{s}=\frac{\left(T_{h}-T_{i}\right)}{R_{h}}+\frac{\left(T_{w}-T_{i}\right)}{R_{wi}}+\frac{\left(T_{f}-T_{i}\right)}{R_{f}}+\frac{\left(T_{a}-T_{i}\right)}{R_{o}}+{\dot{Q}}_{s}$$

where ${\dot{Q}}_{o}$ denotes the transfer of thermal energy to/from the ambient environment through the window,

HTF:

(4) $$M_{h}c_{h}\frac{dT_{h}}{d\tau }={\dot{m}}_{h}c_{h}\left(T_{m1}-T_{m4}\right)+\frac{T_{i}-T_{h}}{R_{h}},\;$$

where

(5) $$T_{h}=\frac{\left(T_{m1}+T_{m4}\right)}{2},$$

wall

(6) $$M_{w}c_{w}\frac{dT_{w}}{d\tau }=-{\dot{Q}}_{wi}+{\dot{Q}}_{wa}=\frac{T_{i}-T_{w}}{R_{wi}}+\frac{T_{a}-T_{w}}{R_{wa}},$$

ceiling/floor:

(7) $$M_{f}c_{f}\frac{dT_{f}}{d\tau }=-{\dot{Q}}_{f}=\frac{T_{i}-T_{f}}{R_{f}}.$$

In the above equations, heat transfer from the liquid zones and surroundings was considered purely convective. In the remaining zones, only conductive heat transfer was taken into account. Thermal resistance between the individual heat exchange surfaces can be written as:

wall–indoor air:

(8) $$R_{wi}=\frac{1}{A_{w}}\left(\frac{1}{\alpha_{i}}+\frac{\delta_{w}}{2\lambda_{w}}\right),$$

wall–surroundings:

(9) $$R_{wa}=\frac{1}{A_{w}}\left(\frac{1}{\alpha_{a}}+\frac{\delta_{in}}{\lambda_{in}}+\frac{\delta_{w}}{2\lambda_{w}}\right),$$

floor/ceiling–indoor air:

(10) $$R_{f}=\frac{1}{A_{f}}\left(\frac{2}{\alpha_{i}}+\frac{\delta_{f}}{\lambda_{f}}\right),$$

cooling ceiling–indoor air

(11) $$R_{h}=\frac{1}{k_{h}A_{h}}.$$

The set of differential Equations (3), (4), (6) and (7) of the considered model can be easily transformed into the normal form (i.e., solved with respect to the differentials). They were solved in real time during the experiment in LabVIEW Control Design (NI, Austin, TX, USA) and Simulation module using the adaptive Runge–Kutta method.

### 2.4. PCM Selection

At the start of the PCM selection process for the LHTES, it should be recalled that water is the most common material for heat storage in hydronic systems for heating, cooling and domestic hot water systems. This is not only due to its favourable thermophysical properties but, above all, to the low costs of its use. When planning the use of the LHTES in a real application, first it should be checked whether its real benefits are greater than in the case of a water tank. This is especially important because of the investment costs as most PCM’s are relatively expensive. Taking this into account, the comparison of the energy accumulated in LHTES with water is particularly important.

The knowledge of thermo-physical properties of heat storage materials is essential in LHTES design. In a case of using PCM, knowledge of the specific heat for both the liquid and solid states is necessary as well as its temperature and specific heat of fusion that is related to the solid–liquid phase transition. These parameters can be often determined using the well-known differential scanning calorimetry (DSC) technique [20,27] Typically, the amount of sampling mass used in DSC is less than 50 mg which can cause many measurement problems. Another way is to use the T-history method, which in some cases gives more reliable results [28]. Based on the known thermo-physical properties, it is possible to estimate the thermal capacity of the storage, but this only applies to the quasi steady-state conditions. As the research shows, the results obtained for a storage with more PCM deviate from the theoretical predictions [17].

**Figure 8:**
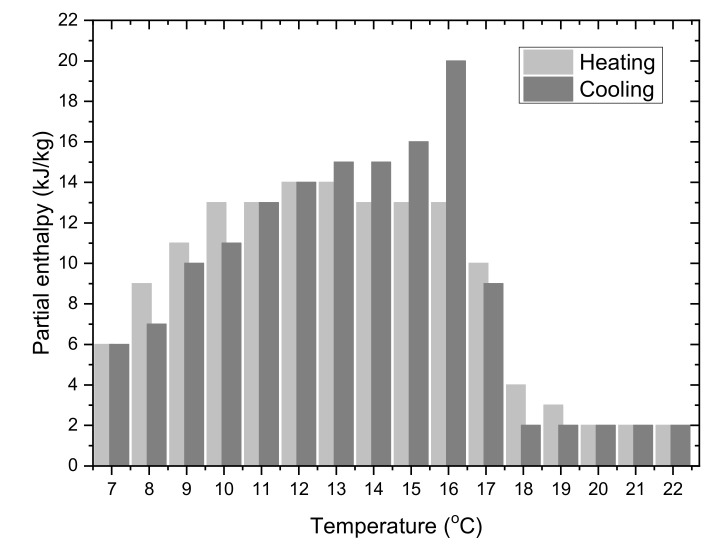
Partial enthalpy distribution as a function of temperature for the phase change material RUBITHERM RT15 [23].

When selecting the PCM, it was initially assumed that the HTF inlet temperature to the cooling ceiling will be 18 °C. This means that the PCM phase transition temperature should be lower than 18 °C. Therefore, the commercially sold PCM named Rubitherm RT15 was chosen. While planning of the commercial implementation of LHTES, the price of PCM should also be considered. The selected material is much cheaper than other organic Rubitherm materials with a similar phase transition temperature but higher heat capacity in a narrower temperature range (the HC-series). Besides low costs, well-documented parameters, cycling stability and low supercooling were the reasons for choosing the RT15. The partial enthalpy distribution for this material, based on manufacturer data, is presented in Figure 8. A summary of its primary thermo-physical properties is presented in Table 3.

It could be seen that the characteristic of the RT15 material provided by the manufacturer does not cover temperatures below 7 °C. At this temperature, the partial enthalpy is higher than 2 kJ/kg. Based on the experiments carried out by the author [17,28], it can be assumed that the phase change process covers a wider temperature range than that presented by the manufacturer.

### 2.5. Experimental Setup

**Figure 9:**
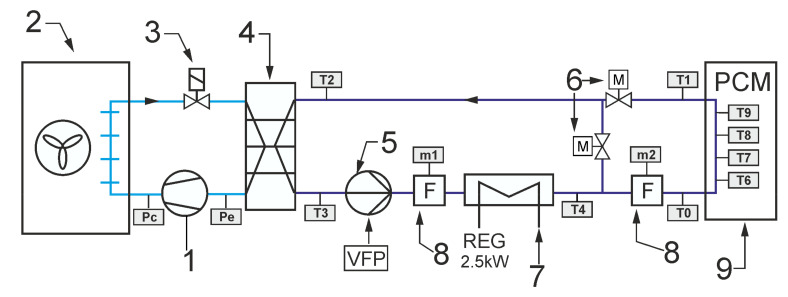
Layout of experimental setup: **1**—compressor, **2**—condenser, **3**—electronic expansion valve, **4**—evaporator, **5**—circulating pump, **6**—control valves, **7**—heater, **8**—flowmeters, **9**—LHTES.

An experimental setup was designed and built to examine dynamic characteristics of different thermal energy storages. Its layout is shown in Figure 9. Two individual loops, separated by the heat exchanger 4 can be distinguished. The first loop (left of Figure 9) is a typical compressor refrigeration cycle. A commonly used thermostatic expansion valve was replaced by an electronic device 3 to attain more stable operation. An algorithm of valve control reduced the fluctuations of the working parameters. During the experiment, the refrigerant parameters were calculated based on the CoolProp library [29]. In the second loop, water circulated as the heat transfer fluid. To ensure fast temperature response of the system, the amount of the water was minimized. Water chilled in the evaporator was pumped to the LHTES container 9 by the variable speed circulating pump 5. A heat transfer fluid flow rate was kept constant by regulating the pump rotating speed. The flow rate in all investigated cases was set to 160 kg/h. The electric heater 7 maintained the set inlet temperature of water. To minimize the fluctuations of this temperature, the heater was equipped with the precise single-phase power controller JUMO TYA-201 (JUMO GmbH, Fulda, Germany). Two coriolis flow meters 8 were used to measure the HTF mass flow rate. E&H Promass 83F DN08 (Endress+Hauser AG, Reinach, Switzerland) mass flow meters with an accuracy of 0.3% were used in the measurements. Danfoss AB-QM (Danfoss A/S, Nordborg, Denmark) control valves 6 with NovoCon digital actuators (Danfoss, Nordborg, Denmark) were used to control the flow. The applied control systems allowed for high precision of experimental conditions.

**Figure 10:**
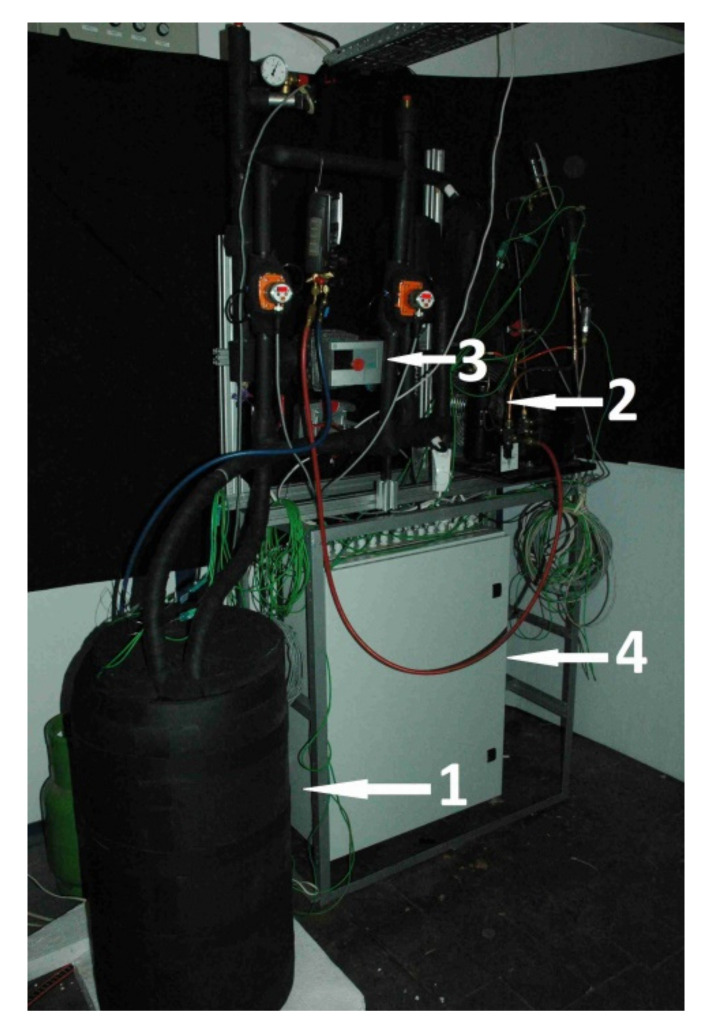
Photo of the experimental stand: **1**—LHTES, **2**—compressor chiller, **3**—circulating pump, **4**—control and measurement system.

K type thermocouple sensors were installed in the key locations of the experimental stand. Two temperature sensors were located at the inlet T0 and outlet T1 of the HTF tube collectors. The temperature from sensor T2, located at the inlet of the evaporator, was used as the input parameter to the mathematical model. Sensor T4, located upstream of LHTES, was used to control the electric heater. Pressure transducers marked as Pe and Pc were used by the refrigerant cycle safety and control system. Four additional thermocouples were installed inside the storage tank between HTF tubes at equal distances of 100 mm to measure the PCM temperature. To attain a fast temperature response, the thermocouple K-type sensors with 1 mm shield diameter were used. All thermocouples were calibrated and tested before installation using the Beamex MC6 calibrator (BEAMEX OY AB, Pietarsaari, Finland), the DRUCK DB-150 (GE-Sensing, Billerica, MA, USA) calibration furnace and the reference temperature sensor PT100 ISOTECH (Isothermal Technology Ltd., Southport, UK). After that, the precision of the thermocouples was ±0.1 °C for 0 °C and ±0.15 °C for 25 °C. Special attention was paid to accurately determine temperature difference between the HTF inlet and outlet of the TES container. The accuracy of this parameter was better than ±0.05 °C. Based on this information, it is possible to estimate the maximum measurement error at ±6% of heat flux and thermal energy measured values. The experimental setup is shown in Figure 10, where the TES container with PCM is visible at the left in the foreground.

Each of the experiments carried out lasted for at least 72 h. For this reason, the real-time controller was used for measurements and to obtain the required experiment parameters. The data acquisition was based on National Instrument Systems and Software. NI cRIO 9014 controller (NI, Austin, TX, USA) was the main part of data acquisition and regulation system. This system logs all key parameters and controls the valves, pumps, electric heaters and safety system. During the experiments, measurement results are shown in real time on the computer screen, and all measured data are saved to a file.

### 2.6. Construction of LHTES Container

**Figure 11:**
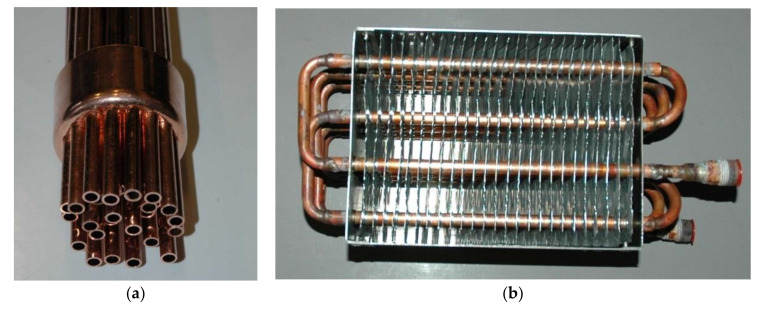
The prototype inserts tested in the shell and tube (**a**) and fin tubes (**b**) versions of the LHTES.

The primary criteria considered during selection of the PCM for a particular application are its phase change temperature and the latent heat capacity. However, other important parameters must also be taken into account in the LHTES design process. First of all, the low thermal conductivity of most of the PCMs (often lower than 1 W/m K) should be taken into account. In thermal storage systems, these low conductivity values may cause problems with reaching the appropriate charging and discharging rates. In order to obtain the suitable thermal power, it is necessary to enhance the heat transfer rate between HCF and PCM by a proper selection of the heat transfer surface. In construction of storage tanks, designs typical for heat exchangers are often used [14,30] Sometimes commercial heat exchangers are adapted for use in PCM thermal storage systems [14]. Due to the complexity of the heat transfer process in LHTES, it is difficult to choose the most important criteria for comparing them. For the purpose of this study, the leading idea in the LHTES design process was to create an inexpensive construction using elements available on the market. Special attention was paid to obtain a large volumetric heat capacity and high dynamics of the loading and unloading process. To form the heat transfer surface between PCM and heat transfer fluid, typical capillary tube mats were used. These mats are applied in housing for wall and ceiling heating and cooling systems [24]. The proposed LHTHS construction was compared with other possible solutions. The shell and tubes, packed bed and a fin tubes designs were chosen for preliminary comparison (some of these constructions previously tested experimentally by the author are shown in the Figure 11).

For comparison, it was assumed that the tank has a cylindrical shape with a diameter of 900 mm and a height of 1800 mm. The influence of edge effects was not considered in the comparison. In all LHTES geometries, due to low typically thermal conductivity of the phase change materials, the key parameter is the ratio of heat transfer area to the PCM volume. The second, but no less important, parameter is the ratio of the PCM volume to the total volume of the vessel. The following three coefficients, based on geometrical parameters, were introduced to evaluate individual constructions of LHTES.

(12) $$G1=\frac{V_{PCM}}{V}\cdot \;(m^{3}/m^{3}),$$

(13) $$G2=\frac{V_{PCM}}{V_{HTF}}\;\cdot \;(m^{3}/m^{3}),$$

(14) $$G3=\frac{A_{HT}}{V}\;\cdot \;(m^{3}/m^{3}),$$

**Figure 12:**
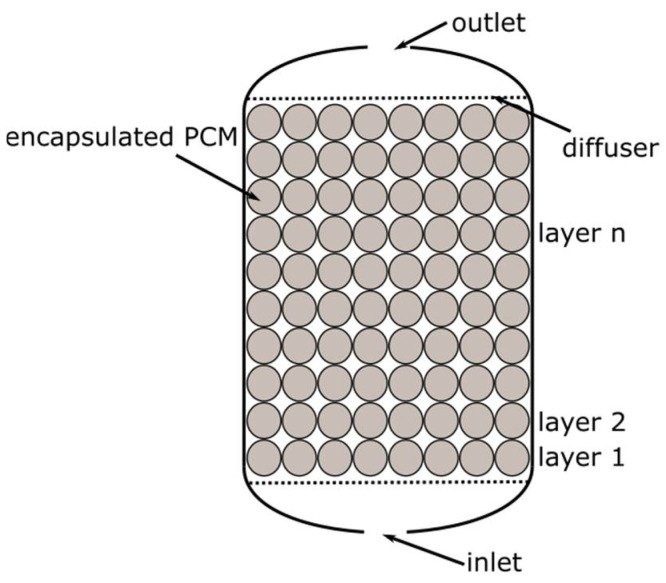
Schematic drawing of the arrangement of the spheres in the vessel.

At first, four packed bed geometries (cases no. 1–4), differing in the capsule diameter, were selected for comparison. It was assumed that:

1. The capsule has a spherical shape;
2. The PCM fills 85% of the capsule volume;
3. The capsules are arranged in layers (Figure 12), and the heat transfer area is the sum of the surface areas of the spheres;
4. Only the volume between the first and the last layer of spheres was considered in the calculations.

The arrangement of the spheres in the vessel is shown in the Figure 12. A summary of the calculated parameters describing the packed bed geometers is presented in Table 4.

Based on previous experiments [31], three shell and tubes geometries, differing in the tubes diameter, were selected for comparison. It was assumed that:

1. Typical copper tubes will be used to build the LHTES (except external housing);
2. The distance between the tube walls is not less than 5 mm;
3. The PCM is placed inside the pipes (cases 5–7);
4. The PCM is placed outside the pipes (cases 8–10).

**Figure 13:**
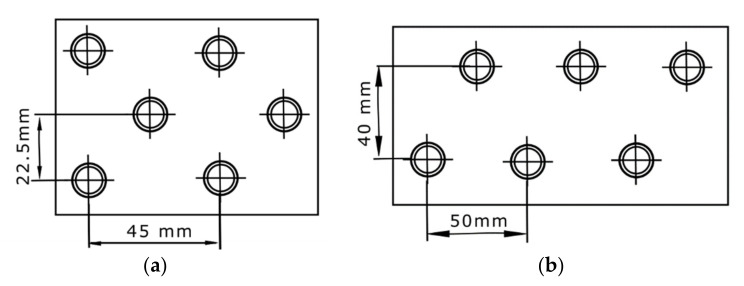
The schematic drawing of fin tubes geometries: (**a**) case 11, (**b**) case 12.

The last geometries chosen for comparison (cases 11 and 12) were based on fin tubes heat exchanger. This type of heat exchanger is used in dry coolers and could be built of elementary blocks. Two geometries offered by the local manufacturer were selected for analysis [32]. They consist of rows of pipes with a diameter of 10 mm, fins of a 0.15 mm thickness and of 14 mm spacing. In this case, the G3 factor (13) was calculated taking into account the surface of the tubes and fins. Selected geometries are shown in Figure 13.

**Figure 14:**
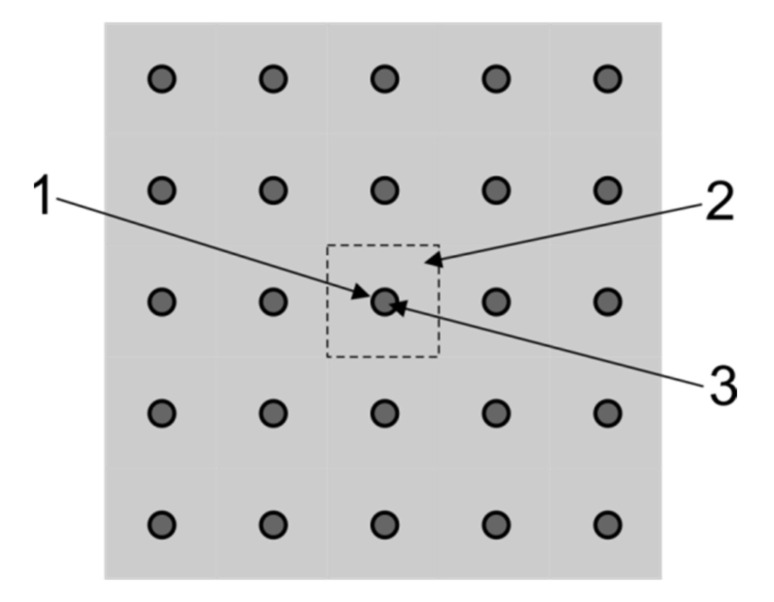
Schematic illustration of the cross-section through the tube bundle and phase-change material (PCM): **1**—tube, **2**—PCM, **3**—heat transfer fluid (HTF). (The dashed line indicates a substitute thickness of the PCM assigned to each tube).

The reference storage geometry (case no. 13) used in comparison with the abovementioned design process is similar to the spiral-shaped coils heat exchanger with PCM on the outside of the tubes. For comparative analysis, the total volume of the PCM is calculated on the basis of the substitute thickness of an adjacent layer assigned to each tube. It was assumed, based on capillary mats technical information, that the diameter of the tubes is 3.35 mm, and the distance between the pipe axes was assumed to be 10 mm. The cross-section of the reference storage geometry is presented in Figure 14. In a storage tank used in the experiments commercially sold capillary tube mats were applied.

**Figure 15:**
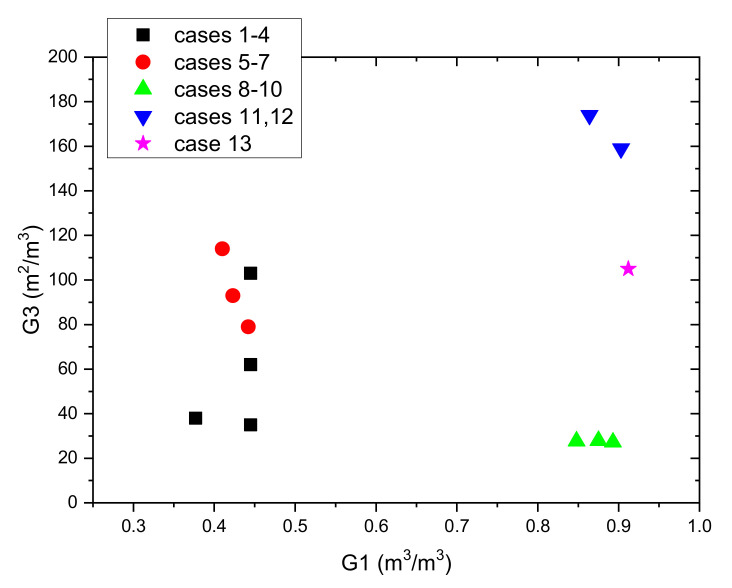
Comparison of analysed LHTES geometries based on G1 and G3 coefficients.

A list of principal geometric parameters and coefficients used for the comparison is presented in Table 4. To help in the comparison of the analysed cases, Figure 15 shows the dependence of the G3 coefficient on G1. It can be seen that the amount of PCM compared to volume of the vessel is the smallest in geometries based on the packed bed. Three of the four such geometries have also the smallest G3 coefficients. This means that the volumetric heat capacity and dynamics of charging and discharging process will be the lowest. The shell and tube geometries (cases 5–7) with PCM filling the tubes have generally a greater G3 coefficient than the packed bed, but also in these cases only half of the vessel volume is filled with PCM. In contrast, the next three cases 8–10, also based on shell and tube geometries, have a very high G1 coefficient. However, the G3 coefficient for these cases is the lowest, which means that the charging and discharging time will be very long. Two geometries based on fin tubes (cases 11 and 12) have the highest G1 and G3 coefficients. This means that both the volumetric heat capacity and charging and discharging velocity is the best among the analysed cases. However, it should be noted that the fin surface was taken into account when determining the heat transfer area in the G3 coefficient. Due to the thermal resistance between the fin and the pipe, the heat exchange efficiency on the fins is much lower. This means that in these cases the G3 coefficient is not adequate to compare the analysed geometries. Additionally, it also means that the real dynamics of the charge and discharge process may be lower. The preferred geometry (case 13), based on the capillary tubes, has the highest G1 coefficient. At the same time, it also has one of the highest G2 and G3 coefficients. Preliminary analysis of the LHTES costs showed that the proposed solution is the cheapest (the packed bed solution costs were not considered). For this reason, a design based on the capillary tubes was chosen for the construction of the LHTES.

**Figure 16:**
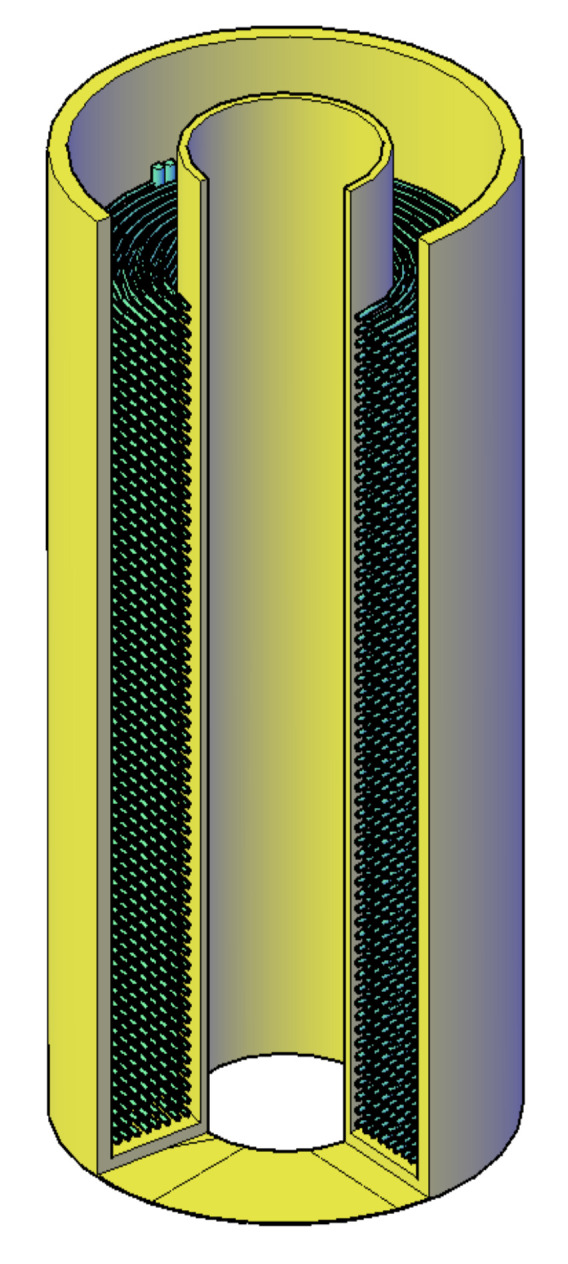
View of LHTES container geometry with capillary mat [17].

Capillary mat BEKA K.G10.6000.0560 (BEKA Heiz- und Kühlmatten GmbH, Berlin, Germany) [24] was chosen for the construction of the LHTES based on the concept presented in the Figure 14. Because of the limited bending radius of the capillary tubes, the usable volume for the PCM is held between two concentric tubes. The internal design of the investigated LHTES container is presented in the Figure 16. The essential parameters of its construction are listed in Table 5. For the designed construction, the ratio of heat transfer area to PCM volume is about 0.15 m^2^/dm^3^. This value is comparable to the best constructions found in literature [14].

**Figure 17:**
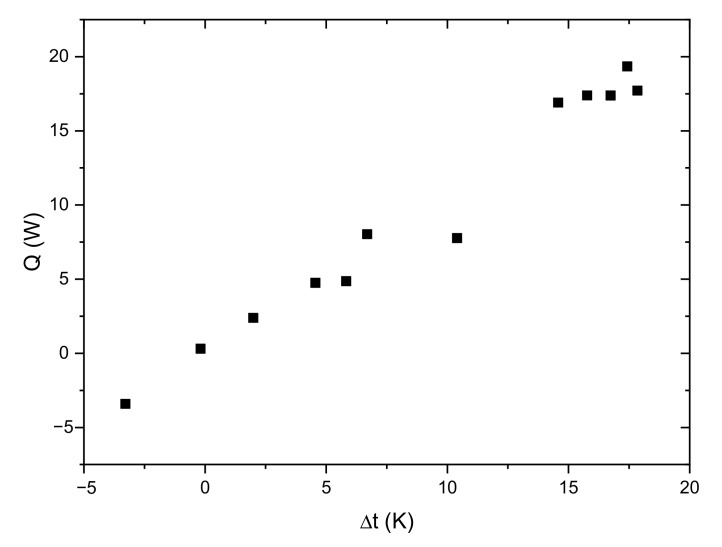
The measured heat flow rate Q as a function of difference between surrounding temperature and average HTF temperature in the steady-state conditions.

Due to the relatively small storage capacity and the long duration of the experiment, the estimation of heat losses to the surroundings is a very important factor. For dynamically operating systems, determining the heat losses is difficult. The instantaneous heat losses can be estimated knowing the ambient temperature and wall temperature of the storage vessel [21]. In this paper, it was assumed that in the analysis only parameters measured in a typical commercial system will be used: ambient temperature as well as the HTF inlet and outlet temperatures. For this reason, a simplified procedure for determining the heat losses to the surroundings was adopted. Steady-state heat flux was determined for various average HTF temperatures (Figure 17). Based on these results, a linear characteristic of the heat losses was determined as a function of the temperature difference between the ambient temperature and the average HTF temperature. Losses to the environment were taken into account during analysis of the measurement results. For the slow-changing processes, it can be assumed that the adopted procedure will be a good estimation of losses to the surroundings.

### 2.7. Data Reduction

The adopted measurement procedure assumes that the LHTES is treated as a black box, which means that it is not possible to measure the internal parameters. This is the case when there is the need to use in the installation a commercially available LHTES with unknown parameters, or the parameters provided by the manufacturer are unreliable. Therefore, at the experimental stand, only the inlet and outlet HTF temperatures as well as the mass flow rate were taken into account in analysis of the measurement results. These three parameters are treated as essential when the operation of the LHTES is to be analysed. PCM temperature is regarded as an auxiliary parameter. This section presents the equations used to obtain the results presented later in the results and discussion section.

The LHTES evaluation is based on the analysis of the instantaneous heat flux and the total heat over one day (24 h) for the charging and discharging process. The instantaneous heat transfer rate between the PCM and HTF was calculated from the following:

(15) $${\dot{Q}}_{PCM}\left(\tau \right)={\dot{m}}_{2}c_{H}\left[T_{1}\left(\tau \right)-T_{0}\left(\tau \right)\right],$$

where ${\dot{m}}_{2}$ is the mass flow rate through the LHTES and *T*_0_ and *T*_1_ are HTF temperatures measured on the LHTES inlet and outlet.

The total heat of charging and discharging process was calculated by means of Equations (16) and (17), respectively:

(16) $$Q_{PCM}^{-}=\sum_{\tau =0}^{\tau =24}{\dot{Q}}_{PCM}\left(\tau \right)\Delta \tau ,$$

where ${\dot{Q}}_{PCM}\left(\tau \right)=0\;$ when ${\dot{Q}}_{PCM}\left(\tau \right)<0$, Δτ=5s,

(17) $$Q_{PCM}^{+}=\sum_{\tau =0}^{\tau =24}{\dot{Q}}_{PCM}\left(\tau \right)\Delta \tau ,$$

where ${\dot{Q}}_{PCM}\left(\tau \right)=0\;$ when ${\dot{Q}}_{PCM}\left(\tau \right)>0,\Delta \tau =5s$.

The instantaneous heat transfer rate to/from the cooling ceiling was calculated by means of the following equation:

(18) $${\dot{Q}}_{h}\left(\tau \right)={\dot{m}}_{1}c_{h}\left[T_{2}\left(\tau \right)-T_{m2}\left(\tau \right)\right]={\dot{m}}_{1}c_{H}\left[T_{m1}\left(\tau \right)-T_{m2}\left(\tau \right)\right],$$

where ${\dot{m}}_{1}$ is the mass flow rate through the cooling ceiling and the evaporator of the refrigerating unit, *T*_2_ is the measured temperature and *T_m2_* is the temperature calculated in the model.

The instantaneous heat transfer rate to/from the ambient through the window was calculated from the following:

(19) $${\dot{Q}}_{o}\left(\tau \right)=A_{o}k_{o}\left[T_{i}\left(\tau \right)-T_{a}\left(\tau \right)\right],$$

where *T_i_* is the indoor temperature calculated from model and *T_a_* is ambient temperature from hourly profile (Figure 4).

The instantaneous heat transfer rate to/from the office wall was calculated using the following relation:

(20) $${\dot{Q}}_{w}\left(\tau \right)=\frac{A_{o}}{R_{iw}}\left[T_{i}\left(\tau \right)-T_{w}\left(\tau \right)\right],$$

where *T_w_* is the wall temperature calculated from model.

Similarly, the instantaneous heat transfer rate to/from the office ceiling/floor was calculated by means of the following equation:

(21) $${\dot{Q}}_{f}\left(\tau \right)=\frac{A_{c}}{R_{f}}\left[T_{i}\left(\tau \right)-T_{f}\left(\tau \right)\right],$$

The instantaneous overall heat transfer rate to/from the office, except the cooling system, was calculated by means of the following equation:

(22) $${\dot{Q}}_{l}\left(\tau \right)={\dot{Q}}_{o}\left(\tau \right)+{\dot{Q}}_{w}\left(\tau \right)+{\dot{Q}}_{f}\left(\tau \right),$$

The total heat within a certain period of time, for all above presented heat transfer rates, was calculated as a sum:

(23) $$Q=\sum_{\tau 1}^{\tau 2}\dot{Q}\left(\tau \right)\Delta \tau ,$$

where, according to the procedure adopted for LHTES (Equations (16) and (17), $Q^{+}$ is only the sum for values $\dot{Q}\left(\tau \right)$ above zero, and $Q^{-}$ is only the sum for values $\dot{Q}\left(\tau \right)$ below zero. From the point of view of temperature changes in the room, heat with a $Q^{+}$ sign increases this temperature, while with a $Q^{-}$ mark, it decreases.

## 3. Results and Discussion

### 3.1. Repeatability and Compliance with the Model

The experimental test stand was designed to carry out the LHTES tests in the cycle corresponding to the operation of the cooling system. The aim was to check whether the use of the additional storage with PCM would allow one to maintain the air temperature within the comfort zone. The operation of the cooling system depends largely on the control algorithm. The model control system has been programmed to perform the following set of tasks after the start of the virtual office building cooling system (Figure 2):

1. Set valve 7 to fully open the flow through the LHTES, set valve 8 to close the flow through the cooling ceiling;
2. Switch ON the circulation pump;
3. After a 10 minutes delay, switch the refrigerating unit ON;
4. The LHTES is charged (chilled) until the HTF temperature reaches 6 °C, and then the refrigerating unit is switched OFF;
5. Set valve 7 to close the flow through the LHTES, set valve 8 to open the flow through the cooling ceiling;
6. At 6 p.m., the refrigerating unit is switched ON;
7. From 6 a.m. to 6 p.m., the PI controller determines the mixing valve position **7** to maintain the indoor temperature *T_i_* in the room in accordance with the set profile *T_set_*;
8. After 6 p.m., set valve 7 to fully open the flow through the LHTES, set valve 8 to close the flow through the cooling ceiling, and thereafter the control cycle repeats.

**Figure 18:**
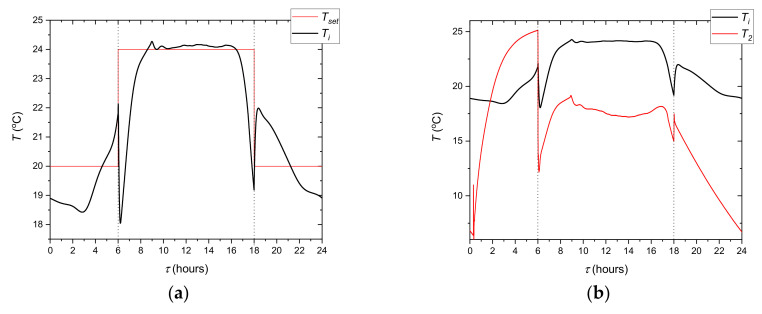
Evolution of the calculated temperature inside of the office *T_i_* (**a**) and the measured inlet HTF temperature *T_2_* (**b**) (cycle no 3).

All elements of the test stand (Figure 9) are controlled to emulate the above-described operation cycle of the cooling system. During the experiments, the circulation pump controller task is to maintain a fixed, set flow rate. It was assumed that the circulation pump would work continuously. In this way it was ensured that there would not be any sudden HTF temperature changes due to the pump starting suddenly. As was mentioned earlier, the step control strategy was tested experimentally (Figure 3). In this strategy, the set temperature rapidly raised from 20 to 24 °C. The PI controller works only from 6 a.m. to 6 p.m. and determines the mixing valve position depending on the difference between the current *T_i_* and the set temperature *T_set_*. It should be remembered that the temperature inside the room *T_i_* is calculated from the mathematical model and is based on the temperature *T_2_* measured before the evaporator (Figure 9). This temperature corresponds to the temperature *T_m1_* in the model. The process for a single cycle may be analysed on the basis of the evolution of temperature *T_i_* (Figure 18). During the night from 6 p.m. to 6 a.m., the indoor temperature in the office is independent of the cooling system state because there is no HTF flow in the cooling ceiling. The model takes this into account through a change of the overall heat transfer coefficient between the cooling ceiling and the air to *k_h_* = 0. At 6 o’clock, the cooling system is started and, due to the small installation volume, the HTF temperature suddenly decreases (Figure 18b). The indoor air temperature also changes rapidly. Such a rapid change results from the low heat capacity of the air and the features of the calculation model used. During the occupied period, the control system maintains the room temperature at the desired level. After 4 p.m., when the heat load decreases, *T_i_* falls below the lower comfort limit. This fall also results from the adopted lumped parameter model and the operation of the PI controller. Namely, the PI controller settings have been optimized to work with relatively slow parameter changes and do not work properly with higher process dynamics. This could be better optimized during further research, but does not significantly affect the obtained results.

**Figure 19:**
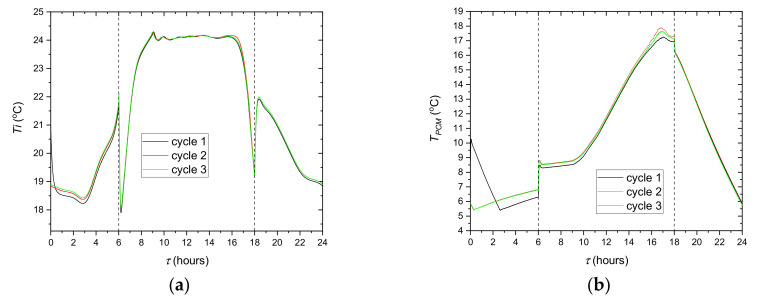
Evolution of the calculated indoor temperature of the office *T_i_* (**a**) and the measured PCM average temperature *T_PCM_* (**b**) in three cycles.

At the beginning of the tests, the whole experimental system usually has an ambient temperature. For this reason, the measurement procedure comprises three cycles of 24 h. Figure 19 shows the temperature repeatability during the three cycles (72 h) with the step cooling system control strategy. Except for the first 6 h of the first cycle, the temperatures follow practically the same trend with deviation lower than ±3%. It can, therefore, be concluded that the presented results show good repeatability after the first cycle. The slight difference between trends 2 and 3 indicates also a small effect of the outside temperature variations. Measurement data from the third cycle are taken for further analysis.

**Figure 20:**
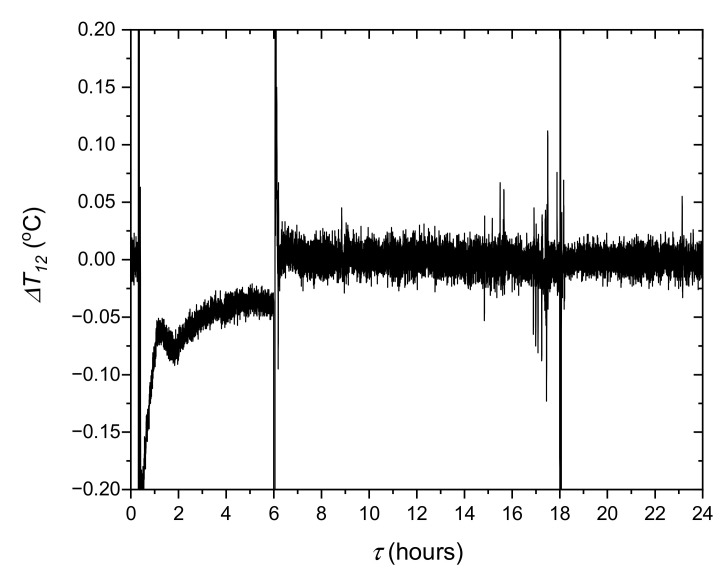
Evolution of the difference between the measured temperature *T*_4_ and *T_m3_* calculated in the model.

Another key issue was to check the mapping of model conditions on the test stand. In the experimental system, the refrigeration device did not have capacity control. For this reason, the temperature was adjusted by means of an electric heater 7 (Figure 8). The PI controller determines the electric power of the heater so that the temperature *T_4_* corresponds to the temperature *T_m3_* calculated from the model. The difference between these temperatures is shown in Figure 20. It can be noticed that the mapping of the model parameters is very good. Apart from the transition states and the period when the control system is off, the difference between temperatures *T*_4_ and *T_m3_* is less than ±0.05 °C. This means that the test stand allows full mapping of the dynamics of the modelled object.

### 3.2. Storage, Charging and Discharging Process

**Figure 21:**
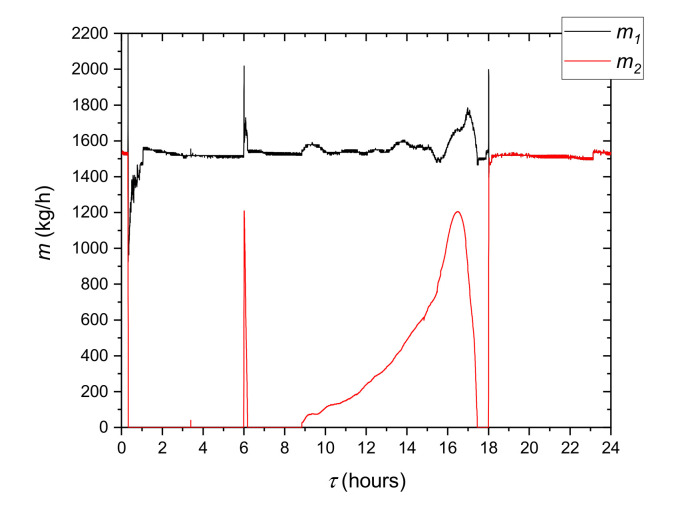
Variation over time of the overall HTF flow rate *m*_1_ and flow rate through the LHTES *m*_2_ during the third cycle.

In the model system during the occupied period, the refrigeration device is always turned on. The cooling system is supported by the LHTES due to insufficient cooling efficiency of the chiller in the on-peak period. The three-way mixing valve 7 is used to control the cooling capacity (Figure 2). On the test stand, this valve is replaced by two throttle valves 6 (Figure 9). The use of such a solution allowed for greater precision of flow control. In Figure 21, the flow rate alteration during the 24 h cycle is shown. It can be observed that the overall HTF flow rate *m*_1_ is kept constant at 1600 kg/h, except for the period from 3 p.m. to 5 p.m. when the flow rate *m*_2_ through the LHTES changes substantially. This causes a significant rise of hydraulic resistance, so the pump flow rate control system cannot keep up with these changes.

**Figure 22:**
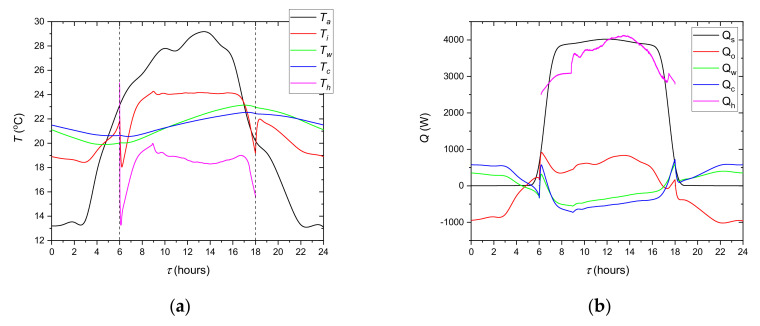
Variation of the temperatures (**a**) and heat transfer rates (**b**) in the modelled office.

**Figure 23:**
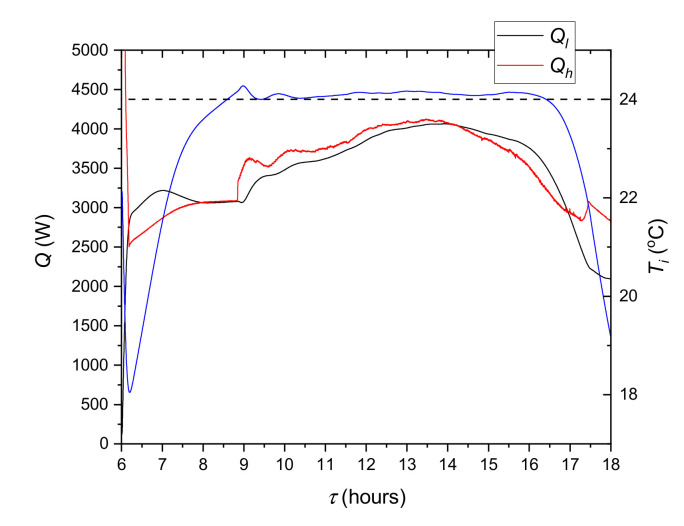
Variation of the indoor temperature *T_i_* (blue line), heat loads *Q_l_* and heat transfer rate *Q_h_* from cooling system. The dashed line indicates the set temperature in the room.

The course of the experiment depends on the thermal behavior of the tested LHTES as well as the modelled system. Just as in a real system, both elements affect each other. The evolution of the modelled temperatures is shown in Figure 22. In addition, the ambient temperature profile is shown there as the main cause of changes in the system. Figure 22b presents the evolution of heat transfer rates corresponding to the temperature profiles. The cooling ceiling temperature *T_h_* and the corresponding heat transfer rate *Q_h_* are presented only from 6 a.m. to 6 p.m. This is due to the absence of the HTF flow through the cooling ceiling during the remaining time. It can be seen that the *T_i_* decreases from 0 a.m. to about 3 a.m.: the reason being that the *Q_o_* is lower than the sum of *Q_w_* and *Q_c_*. The same temperature drop occurs after 6 p.m. During the occupied period, *T_i_* is stabilized in the comfort zone by the cooling system. Building elements also stabilize the temperature, making its changes slower. Analysis of Figure 20 indicates also that the main component of heat load in the occupied period is *Q_s_*. During this time building elements have a lower temperature than air and tend to decrease temperature in the office. The period from 6 a.m. to 6 p.m. is detailed in Figure 23. It can be seen that the cooling system keeps up with changes in heat load and stabilizes the inner temperature at 24 °C. It can also be seen that the air temperature was lower than 20 °C in the periods with reduced demand for cooling capacity. It was caused by too high a capacity of the refrigeration system which was adjusted to the maximum demand. This can be corrected by changing the control algorithm; however, this complicates the system. Such modifications will be taken into account in further research.

**Figure 24:**
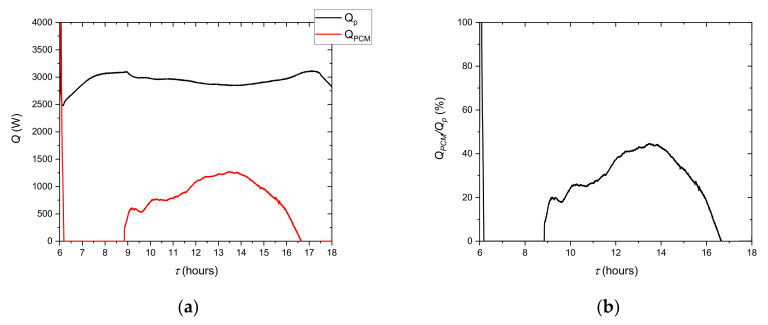
Variation of the LHTES and chiller heat transfer rates during the occupied period (**a**) and the percentage ratio of the heat transfer rates (**b**).

From the point of view of the cooling system evaluation, it is important to determine the effect of the LHTES. As it can be seen in Figure 5, using only a chiller will not keep the air temperature in the comfort zone. Figure 24 shows the variation of the LHTES and chiller heat transfer rates during the occupied period. It can be observed (Figure 24b) that the LHTES provides coverage of the cooling demand during the peak period as high as 42%. The dynamics of the LHTES discharge process allowed the air temperature to be maintained at the set level.

Heat values accumulated in or discharged from the LHTES and office room elements included in the model are presented in Table 6 and Table 7. For the office space, the energy balance over the entire cycle (24 h) can be represented as the following:

(24) $$Q_{h}^{-}+Q_{w}^{-}+Q_{f}^{-}+Q_{o}^{-}-Q_{s}^{+}-Q_{w}^{+}+Q_{f}^{+}+Q_{o}^{+}=\Delta Q_{ofice},$$

where $\Delta Q_{ofice}$ is the unbalanced heat.

Heat delivered to the room throughout the whole day is 59.2 kWh and received 57.2 kWh. The difference of 2 kWh is caused by several reasons, but is most likely due to measurement uncertainties of different parameters involved in the analysis, unbalance of the test stand and overall heat losses. To summarize, it can be stated that the imbalance at 3.5% for a 24 h experiment is small. Considering the cooling system, energy balance over the entire cycle (24 h) can be presented as the following:

(25) $$Q_{e}^{-}+Q_{PCM}^{-}-Q_{h}^{-}-Q_{PCM}^{+}=\Delta Q.$$

The thermal energy obtained from the refrigeration system and LHTES during the discharge is 58.4 kWh. The energy consumed by the receiver and LHTES during charge cycle is 56.8 kWh. In these cases, unbalancing is 1.6 kWh, and it can be considered acceptable. It can be seen that for the whole day, the thermal energy obtained from the process of discharging the LHTES constitutes 15.5% of the energy obtained from the refrigeration device. A significant difference can also be observed between the energy supplied in the charging process and that received in the unloading process of the LHTES. That difference is 7.3 kWh and can be caused by many factors including energy losses to the surroundings and the effect of subcooling. Comparing the obtained thermal energy to the theoretical value (see Table 8) resulting from the change in HTF temperature, it can be seen that 33.4% less energy was obtained. This is related to the heat transfer process in the LHTES and indicates a high non-homogeneity of the phase change process in its volume. Such a difference also indicates that in the real LHTES, there are many areas in which the PCM is far away from the heat transfer surface or the so-called dead zones.

It can be seen that for the adopted boundary conditions, the energy $Q_{h}^{-}$ received by the cooling system is comparable to the energy of internal sources $Q_{s}^{+}$. This is due to the fact that in daily cycles the average surrounding temperature is close to the average indoor temperature (see Table 6). The convergence of these temperatures is accidental but for subsequent experiments a wider range of parameters should be taken into account. Structural elements such as walls and ceilings are significant elements that accumulate heat and reduce the temperature fluctuations inside the room. When optimizing the cooling system, these elements should be included in the calculations.

The cooling and storage system is designed to keep the room temperature at the right level. It can be seen (Figure 22b) that the temperature was kept at 24 °C during peak hours. During this period, 22% of the energy came from LHTES discharge. At some moments (Figure 24b), over 40% of the heat energy came from LHTES. Due to insufficient capacity, the refrigeration system alone would not ensure that the temperature is maintained in the comfort zone. Concerning to the capacity of LHTES, it can be noticed that the theoretical heat storage capacity of PCM (see Table 8) is about 38% higher than that obtained in this research (see Table 6). As it was already mentioned, it depends on the dynamics of the charging and discharging process. It can be supposed that the temperature change was too abrupt to obtain the desired level of homogenisation in the storage tank.

## 4. Conclusions and Recommendations for Future Work

This paper evaluates how to verify the thermal energy capacity and the charging/discharging rate of a thermal energy storage tank in real-like conditions. To achieve this goal:

1. The thermal behaviour of the LHTES was tested based on the inlet and outlet HTF temperatures and flow rates. The adopted measurement procedure assumes that it is not possible to measure the internal temperature of the PCM, and the LHTES is treated as a black box. For example, this type of approach is essential when a commercially available LHTES with unknown parameters should be tested.
2. During the experiment, the HTF temperature at the LHTES inlet was calculated based on the simulation of the office building with the cooling system.
3. A lumped parameter model was used to describe mathematically the office building and its cooling system. The simulations were made with the measured hourly ambient temperature profile and assumed office building internal cooling load.
4. The elements of the stand allowed to emulate the overall calculated heat load. It can also be observed that the assumed solution of the test stand allows full mapping of the dynamics of the modelled object.

In the author’s opinion, such an approach can provide reliable information about the storage capacity of the LHTES and the dynamic characteristic during the loading and unloading process. Selecting of the case with the highest energy demand for testing ensures that the storage will perform well under other external conditions. This method also allows the optimization of the settings of the control system under dynamic conditions corresponding to real thermal loads.

Based on the test procedure run, some suggestions can be made for improving research methodology:

1. Firstly, in some situations the mathematical model of the office building provided unphysical results. For example, after 6 a.m. the indoor air temperature changes rapidly. This significantly influences the regulation process and destabilises the system operation. In the case of this type of experimental research, an excessive complication of the model is not recommended, but such problems should be removed.
2. The next change concerns the control of the circulation pump. It was assumed that the circulation pump would work continuously to prevent sudden HTF temperature changes. This resulted in a continuous increase in HTF temperature in the storage period (from 0.30 a.m. to 6 a.m.). Due to the small volume of water, the heat supplied by the pump during this time caused an error in the heat balance. In industrial systems, in such a situation pump is switched off and the same procedure should be implemented in research methodology.
3. Except for the evaluation of the experimental methodology, the second goal of the research was to verify a prototype construction of LHTES filled with PCM suitable for the studied heat load case. The following conclusions can be drawn from this part of the research:

Regarding the HTF temperature range (Table 7), it can be noticed that the chiller works with low efficiency in the charging period. The main reason for this is the selection of a PCM with a very wide effective specific heat distribution. To improve the efficiency of the system with the PCM tanks using the cooling ceiling, more optimal material selection is required.

It can be observed from the comparison of the stored thermal energy to its theoretical value that 33.4% less energy was obtained. This is related to the heat transfer process in the LHTES and indicates a high non-homogeneity of the phase change process in its volume. Such a difference also shows that in the real LHTES, there are many areas in which the PCM is too far away from the heat transfer surface in the so-called dead zones. The influence of these areas is particularly evident for small PCM tanks and indicates that in future work experimental studies on a larger scale are needed.

The analysis of the obtained results allows for a general conclusion concerning the residential cooling systems supported by the PCM tanks. In real systems, the difference between the heat transfer fluid temperature and PCM is typically lower than in the most of the tests presented in the literature. The presented approach, based on an idea of experimental testing of the operation of LHTES in real-like conditions, could be a useful solution for estimation of the thermal capacity of the LHTES. Moreover, the adopted methodology allows to determine an adequate dynamic characteristic of the storage PCM tanks.

## Figures and Tables

**Table 1:** The coefficients of polynomial (1) for capacity and the electric power of BITZER 2KES-05Y compressor.

Polynomial Coefficients	Cooling Capacity	Electric Power
*c*_1_	3082.922252	320.8645426
*c*_2_	120.5396381	2.694097539
*c*_3_	−40.92334544	4.054368978
*c*_4_	1.723510406	0.091223069
*c*_5_	−0.870318216	0.106588089
*c*_6_	0.267785529	0.108003543
*c*_7_	0.008886353	0.001186798
*c*_8_	−0.009993387	−0.003202868
*c*_9_	−0.000394424	0.001282039
*c*_10_	−0.001333982	−0.000958283

**Table 2:** Detailed information about the elements included in the office model.

Parameter	*A* (m^2^)	*d* (m)	*ρ* (kg/m^3^)	*c* (J/kgK)	*γ* (W/mK)	*α* (W/m^2^K)	*k* (W/m^2^K)
wall	72	0.29	800	800	0.3	–	–
ceiling/floor	100	0.18	2300	840	0.3	–	–
windows	48	–	–	–	–	–	1.1
cooling ceiling	100	–	–	–	–	–	–
insulation	72	0.1	–	–	0.045	–	–
indoor air (i)	–	–	1.2	1000	–	5	–
ambient air (a)	–	–	1.2	1000	–	10	–
HTF	–	–	1000	4200	–	–	6.5

**Table 3:** Summary of the primary thermo-physical properties of RUBITHERM RT 15 [23].

Parameter	Value
melting area	10–17 °C
congealing area	17–10 °C
heat storage capacity (latent and sensible heat)	150 kJ/kg
specific heat capacity (both phases)	2 kJ/kg
density solid (at 15 °C)	880 kg/m^3^
density liquid (at 20 °C)	770 kg/m^3^
heat conductivity (both phases)	0.2 W/mK
volume expansion	12.5%

**Table 4:** Summary of the parameters describing the analysed geometers.

Case No.	d (mm)	n ^1^	g ^2^ (mm)	G1	G2	G3
1	30	54,000	0.5	0.445	0.933	103
2	50	11,660	0.5	0.445	0.933	62
3	70	3600	0.5	0.377	0.678	38
4	90	2000	0.5	0.445	0.933	35
5	15.9	1846	0.4	0.410	0.748	114
6	22	1089	1	0.423	0.868	93
7	28	729	1.5	0.442	0.995	79
8	15.9	1846	0.4	0.893	9.15	27.2
9	22	1089	1	0.875	8.80	27.9
10	28	729	1.5	0.848	6.74	27.7
11	10	−	0.5	0.864	13.1	173.9
12	10	−	0.5	0.903	27.1	158.9
13	3.5	−	0.5	0.912	21.0	105

^1^ Number of spheres/tubes in the LHTES geometries; ^2^ Wall thickness.

**Table 5:** Summary of principal geometrical and thermo-physical properties of the LHTES container.

Parameter	Unit	Value
Capillary tube material		polypropylene
Capillary tube outer diameter	mm	3.35
Capillary tube wall thickness	mm	0.5
Capillary tubes spacing	mm	10
Capillary tube length	mm	6000
Spacing between tubes layers	mm	10
Capillary tube material thermal conductivity	W/m·K	0.24 [18]
Capillary tube material specific heat	J/kg·K	2000 [18]
Capillary tube mass	kg	1.35
Container material		polyethylene
Container mass	kg	13.1
Insulation material		Armacell Armaflex ACE Plus
Insulation thickness	mm	30
HTF mass	kg	1.5
Heat transfer area	m^2^	4.097

**Table 6:** Summary of 24 h cycle heat values.

Parameter	Unit	Value
$Q_{PCM}^{-}$	kWh	7.8
$Q_{PCM}^{+}$	kWh	15.1
$Q_{s}^{+}$	kWh	42.9
$Q_{e}^{-}$	kWh	50.6
$Q_{w}^{-}$	kWh	3.6
$Q_{w}^{+}$	kWh	3.3
$Q_{h}^{-}$	kWh	41.7
$Q_{c}^{-}$	kWh	5.2
$Q_{c}^{+}$	kWh	5.2
$Q_{o}^{-}$	kWh	6.7
$Q_{o}^{+}$	kWh	7.8

**Table 7:** Summary of heat values during the occupation period (6 a.m.–6 p.m.).

Parameter	Unit	Value
$Q_{PCM}^{-}$	kWh	0.1
$Q_{PCM}^{+}$	kWh	7.8
$Q_{s}^{+}$	kWh	42.5
$Q_{e}^{-}$	kWh	35.2
$Q_{w}^{-}$	kWh	3.4
$Q_{w}^{+}$	kWh	0.4
$Q_{h}^{-}$	kWh	5.2
$Q_{c}^{-}$	kWh	0.5
$Q_{c}^{+}$	kWh	0
$Q_{o}^{-}$	kWh	6.5
$Q_{o}^{+}$	kWh	0.1

**Table 8:** Summary of important results obtained during the third cycle (24 h) of the experiment.

Parameter	Unit	Value
Average surrounding temperature	°C	21.3
Average indoor temperature	°C	21.6
Minimum of average HTF temperature	°C	5.2
Maximum of average HTF temperature	°C	18.6
Minimum of average PCM temperature	°C	5.4
Maximum of average PCM temperature	°C	17.6
Theoretical heat storage capacity of PCM ^1^	kWh	10.8
Theoretical heat storage capacity of PCM ^2^	kWh	10.7

^1^ Based on the PCM characteristics (Figure 8) and variation of average HTF temperature. ^2^ Based on the PCM characteristics and variation of average PCM temperature.

## Data Availability

The data presented in this study are available on request from thecorresponding author.

## Nomenclature and Abbreviatiaons

**Nomenclature**	
*A*	surface area (m^2^)
*c*	specific heat (J/kg·K)
Δ*h_SL_*	latent heat (J/kg)
*k*	overall heat transfer coefficient (W/m^2^·K)
*l*	length (m)
$\dot{m}$	mass flow rate (kg/s)
*M*	mass (kg)
*R*	thermal resistance (K/W)
*E*	thermal energy (J) (kWh)
$\dot{Q}$	heat flow rate (W)
*T*	temperature (K) (°C)
*α*	heat transfer coefficient (W/m^2^·K)
*τ*	time (s) (h)
*Subscripts*	
1	*inlet*
2	*outlet*
*a*	surroundings
*c*	condensation
*e*	evaporation
*f*	floor, ceiling
*h*	cooling ceiling
*i*	indoor air
*in*	insulation
*m*	modelled parameter
*τ*	time (s) (h)
*o*	window
*w*	wall
**Abbreviations**	
EER	energy efficiency ratio
HTF	heat transfer fluid
ITES	ice thermal energy storage
LHTES	latent heat thermal energy storage (with PCM)
PCM	phase change material
PI	proportional-integral controller
TES	thermal energy storage
